# *In Vivo* Monitoring of mRNA Movement in *Drosophila* Body Wall Muscle Cells Reveals the Presence of Myofiber Domains

**DOI:** 10.1371/journal.pone.0006663

**Published:** 2009-08-17

**Authors:** Alice M. C. van Gemert, Annelies M. A. van der Laan, Gonneke S. K. Pilgram, Lee G. Fradkin, Jasprina N. Noordermeer, Hans J. Tanke, Carolina R. Jost

**Affiliations:** 1 Laboratory of Gene Expression and Imaging, Department of Molecular Cell Biology, Leiden University Medical C, Leiden, The Netherlands; 2 Laboratory of Developmental Neurobiology, Department of Molecular Cell Biology, Leiden University Medical C, Leiden, The Netherlands; German Cancer Research Center, Germany

## Abstract

**Background:**

In skeletal muscle each muscle cell, commonly called myofiber, is actually a large syncytium containing numerous nuclei. Experiments in fixed myofibers show that mRNAs remain localized around the nuclei in which they are produced.

**Methodology/Principal Findings:**

In this study we generated transgenic flies that allowed us to investigate the movement of mRNAs in body wall myofibers of living *Drosophila* embryos. We determined the dynamic properties of GFP-tagged mRNAs using *in vivo* confocal imaging and photobleaching techniques and found that the GFP-tagged mRNAs are not free to move throughout myofibers. The restricted movement indicated that body wall myofibers consist of three domains. The exchange of mRNAs between the domains is relatively slow, but the GFP-tagged mRNAs move rapidly within these domains. One domain is located at the centre of the cell and is surrounded by nuclei while the other two domains are located at either end of the fiber. To move between these domains mRNAs have to travel past centrally located nuclei.

**Conclusions/Significance:**

These data suggest that the domains made visible in our experiments result from prolonged interactions with as yet undefined structures close to the nuclei that prevent GFP-tagged mRNAs from rapidly moving between the domains. This could be of significant importance for the treatment of myopathies using regenerative cell-based therapies.

## Introduction

The movement of mRNAs within the cytoplasm is central to the regulation of translation. Directed movement of a subset of mRNAs to distinct sites within the cell allows for local translation. This process is common in highly polarized cells such as oocytes or neurons and is essential to maintain the morphology of these cells [1].

The movement of mRNAs in the cytoplasm is not restricted to the directional movement of specific mRNAs that need to be locally translated. Movement of mRNAs is also an essential aspect of mRNA metabolism with mRNAs being in dynamic flux between different subcellular locations depending on the fate of the mRNAs. During translation mRNAs are associated with polysomes while mRNAs that are targeted for degradation move to processing bodies (P-bodies) [2]. Finally mRNAs that need to be translationally repressed associate into stress granules [3]. The differential distribution of the mRNAs between the above mentioned structures determines the rate at which mRNAs are degraded or translated and ultimately determines the level of protein production. This view on mRNAs is in agreement with the finding that single mRNA molecules diffuse through the cell but can also be transiently anchored or actively transported [4].This holds true for all cytoplasmic mRNAs, both the targeted mRNAs that are directed to a specific location within the cell and the mRNAs that move as part of the normal process of metabolic regulation [4], [5]. It was therefore proposed that both types of mRNA move by a common mechanism but that the localized mRNAs bind more frequently to transport structures and travel longer distances.

While the dynamics of mRNAs within mononuclear cells has been documented, the movement of mRNAs in myofibers has not yet been addressed. Indeed mRNA mobility in these cells might well be more complex as myofibers are large multi-nucleated cells in which each nucleus is thought to produce mRNAs for a limited area of cytoplasm [6].The best studied example of compartmentalized gene expression in muscles involves proteins that specifically localize to the neuromuscular junction (NMJ) [7]. These proteins are produced as a result of a specific gene expression program that is active in the nuclei clustering immediately under the NMJ [8], [9]. For this nuclear heterogeneity to result in local mRNA translation the mRNAs must either be specifically targeted to the NMJ or mRNA mobility in general must be limited in myofibers. Studies suggest that both mechanisms are employed in myofibers. For example utrophin mRNAs contain sequences that preferably target them to the NMJ [10] and Ralston and Hall have shown that three different mRNAs encoding proteins that localize to the nucleus, the cytoplasm or the endoplasmic reticulum remain confined to the area around the nucleus from which they originate [11]. In this study we ask if the movement of mRNAs within the myofibers is indeed constrained and address how the restricted mobility is brought about. Towards this end we have studied the spatiotemporal dynamics of mRNAs in myofibers of living *Drosophila* embryos.

We studied myofibers at stage 16 of *Drosophila* embryo development. Each embryo segment contains 30 well-described multinucleated somatic muscle fibers, which are attached to the body wall. At stage 16, the muscles are innervated [12] but do not contract yet due to the absence of motoneuron electrical activity. At this stage the embryos are relatively small, thus, permitting the visualization of fluorescent markers using confocal laser scanning microscopy in living animals without dissection. Experiments were performed in muscles 8 and 12, both large muscles located relatively close to the body wall.

To visualize mRNA movement in living embryos we used the MS2-system [13] that allows an artificial reporter mRNA to be fluorescently labelled *in vivo*. The reporter mRNA contained the SV40 3′UTR (Untranslated Region) that is commonly present in *Drosophila* transgenes to increase mRNA stability [14]. This 3′UTR does not contain any known localization sequences and the behaviour of this mRNA is therefore likely representative of non-targeted mRNAs. We chose this approach because our aim was to investigate constraints to mRNA mobility independent of signalling sequences.

To date the restricted movement of mRNAs in myofibers had been deduced from the localization of mRNAs that originated from a specific nucleus in fixed cultured hybrid myofibers. The mechanism by which the mobility was restricted remained elusive. In this study we visualised the dynamic behaviour of non-targeted reporter mRNAs in myofibers of living *Drosophila* embryos and show that the fibers consist of domains. The movement of the mRNAs within the domains is fast but exchange between the domains is relatively slow. Our data therefore suggest that mRNAs undergo prolonged interactions to as yet unknown structures present at the domain boundaries.

## Results

### GFP-tagged mRNAs in the cytoplasm of myofibers of living Drosophila embryos

**Figure 1:**
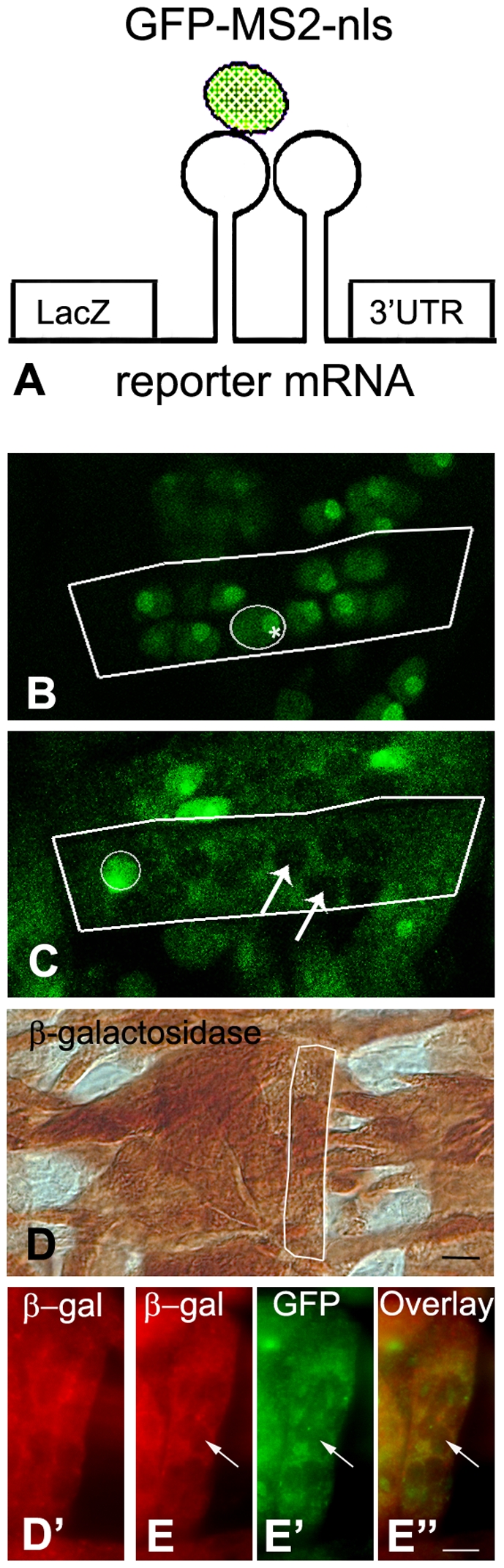
A binary system for in vivo monitoring of mRNA mobility. (A) System components, reporter mRNA with MS2 binding-sites and GFP-MS2-nls fusion protein. (B–C) Maximum projections of z-series of embryonic muscles: distribution of GFP-MS2-nls in *Drosophila* myofibers in the absence (B) or presence (C) of the reporter mRNA. Muscles 12 are outlined in white. Circles indicate nuclei containing the GFP-fusion protein. Note heavily labelled nucleoli (*). (C) In the presence of the reporter mRNA, GFP-MS2-nls is observed in the cytoplasm. Nuclei either contain or lack GFP-MS2-nls protein. High levels of expression result in the accumulation of GFP-fusion protein in the nuclei. Arrows indicate empty nuclei. (D, D') DAB-peroxidase immunohistochemistry and immunofluorescence showing the presence of ß-galactosidase in the absence of the GFP-fusion protein. (E–E'') The presence of ß-galactosidase (red) in muscle 12 expressing the GFP-fusion protein (green). E'' shows overlay. Bar (E''), 5 µm.

To study the movement of mRNAs in *Drosophila* embryonic muscle fibers we generated transgenic flies directing the synthesis of the reporter mRNA and a Green Fluorescent Protein (GFP)-fusion protein which are the two components of the MS2 tagging system (Fig. 1A). GFP was fused to the coat protein of the MS2 bacteriophage which binds mRNA stem-loops. The GFP-MS2 construct also contains a Nuclear Localization Signal (nls) in order to direct the GFP-fusion protein to the nucleus when expressed alone or unbound to the reporter mRNA. The second construct is a reporter mRNA which consists of eight binding sites (19-base stem-loop) for the MS2 coat protein, a ß-galactosidase coding sequence to monitor expression of the construct and the SV40 3′UTR (Untranslated Region) to mimic the behaviour of non-targeted mRNAs. The UAS-GAL4 system developed by Brand and Perrimon [15] is used to induce ectopic expression of these transgenes in muscle. Therefore both constructs contain a UAS promoter that is activated by GAL4. Their expression is driven by the 24B-GAL4 driver which induces GAL4 expression during the early phases of mesoderm development and maintains expression in the musculature throughout embryogenesis.

Newly transcribed reporter mRNA is bound in the nucleus by the GFP-fusion protein and the formed complex exits the nucleus and is found in the cytoplasm. The *in vivo* affinity of GFP-MS2-nls for RNA containing MS2 binding sites is high (K_on_/K_off_ = 500) [16]. This is in agreement with in vitro dissociation kinetic measurements performed with this mRNA-MS2 complex that show that the half life of this complex is 6 hours [17] Therefore, GFP-MS2-nls remains tightly associated with the reporter mRNA during the course of our experiments. During cell division and concomitant nuclear envelope breakdown, the fusion protein could also be present in the cytoplasm of cells in the absence of the reporter mRNA. However, as muscle fibers are post-mitotic, this does not occur in our experiments. The ratio between the two constructs is crucial for correct functioning of the system as excess GFP-fusion protein that does not bind to the reporter mRNAs accumulates in the nucleus. To minimize nuclear accumulation of GFP we used the fly lines expressing the lowest visible level of GFP-fusion protein and the highest level of reporter mRNA, as determined by a ß-galactosidase activity assay (data not shown).

As expected, when the GFP-fusion protein was expressed alone it localized in the nuclei (Fig. 1B), with the fluorescence intensity in the nucleus being 5.5 times as high as in the cytoplasm (n = 11). The ratio between the fluorescence signal in the cytoplasm and the background is 2,36 (n = 11), showing that little GFP-fusion protein remains present in the cytoplasm. We also found that the GFP-fusion protein in the absence of the reporter mRNA preferentially localizes in the nucleoli (Fig. 1B, asterisk), suggesting that GFP-MS2-nls also binds ribosomal RNAs that are produced in that area of the nucleus. This might be due to the affinity of the MS2 coat protein for stem-loop structures that are present in rRNAs. If this binding indeed occurs we assume that the affinity of the fusion protein for ribosomal RNAs in the cytoplasm is low as this binding is insufficient to retain the nls-containing fusion protein in the cytoplasm and will not interfere with our measurements in the cytoplasm. In the presence of the reporter mRNA, the GFP-fusion protein relocates to the cytoplasm, leaving the nuclei empty (Fig. 1C). GFP exits the nucleus together with the reporter mRNA and can be visualized throughout the cytoplasm of the muscle cell under investigation. The ratio between the fluorescence signal in the cytoplasm and the background is doubled (4,66; n = 52) when compared to the situation where the GFP-fusion protein is expressed alone (ratio 2,36). We observed considerable variation in the amount of GFP present in the nuclei. Some nuclei lacked GFP (Fig. 1C, arrows), while others contained a substantial amount of GFP (Fig. 1C, white circle). To date, we are unable to explain this variation between nuclei of the same muscle. As multiple GFP-fusion proteins can be translated from a single mRNA, the ratio between the reporter mRNA and the GFP-fusion protein becomes skewed over time towards an excess of GFP. As a result all nuclei become GFP positive as the embryo further develops. Our experiments were performed in myofibers containing at least a few empty nuclei. Note that there is a slight accumulation of GFP-fusion protein at the transcription loci of the reporter mRNA, resulting in a small spot visible inside the empty nuclei [18].

To verify that the expression of the GFP-fusion protein does not hinder the nuclear export of the reporter mRNA we examined whether translation of the reporter mRNA takes place when the GFP-fusion protein is present. We detected ß-galactosidase in the myofibers both in the absence (Fig. 1D, D') and presence of the GFP-fusion protein (Fig. 1E–E'') by immunolabeling. Together, this indicated that the translation of reporter mRNAs takes place in the presence of the GFP-fusion protein and that the GFP-fusion protein does not retain the reporter construct in the nucleus. Interestingly, a quantitative β-galactosidase assay performed on *Drosophila* larvae showed that the translation of the reporter mRNAs in myofibers is consistently increased in the presence of the GFP-fusion protein (data not shown). This further supports our finding that the fusion protein does not retain the reporter mRNA in the nucleus.

### Myofiber domains revealed by mRNA movement

**Figure 2:**
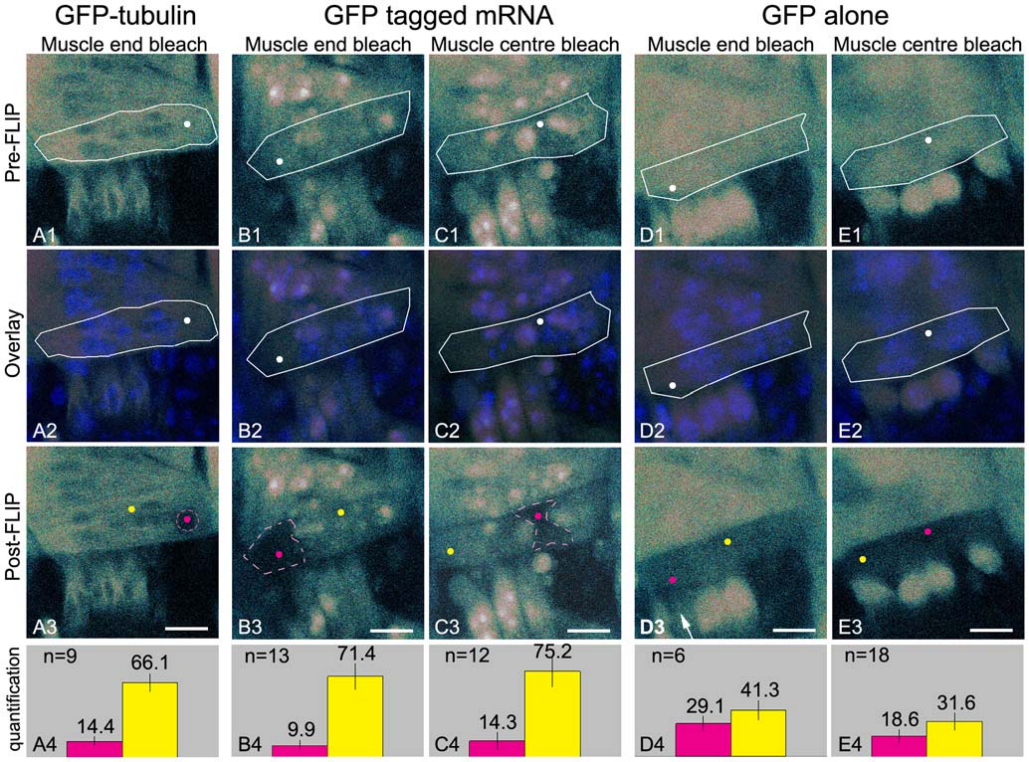
The movement of GFP-tagged mRNAs in muscle 12 reveals myofiber domains. (A1–E1) Pre-FLIP confocal images. White dots indicate the bleach spot and white lines indicate the circumference of muscle 12. (A2–E2) Overlay of the pre-FLIP images and confocal images showing Hoechst stained nuclei. This nuclear staining is used to ensure that either bleach spot (white dot) or quantification areas are chosen outside of the nuclei (A3–E3) Post-FLIP confocal images. Muscle cells were submitted to 20 cycles of two frames and ten bleach events. Pink and yellow dots indicate the location in which quantification was performed before and after the FLIP experiment. Pink dots were located at the bleach spot, yellow dots were positioned in the domain adjacent to the bleached domain. (See below for explanation of the bleached domain). (A4–E4) Quantification. Bar graphs give the percentage of fluorescence remaining within the bleach spot (pink dots A3–E3) in pink and the percentage remaining in the domain adjacent to the bleached domain (yellow dots A3–E3) in yellow. Error bars represent the standard deviation. Bars (A3–E3), 8 µm. (A1–A4) Myofibers expressing GFP-tubulin (bleaching in the muscle end). (A3) Fluorescence is lost in a discrete area of 4 µm surrounding the bleach spot. (A4) Bar graph shows that fluorescence mostly disappears from the bleach spot (pink) and that the fluorescence outside the bleached domain (yellow) is only slightly diminished. (B1–B4) Myofibers expressing GFP-tagged mRNA (bleaching in the muscle end). (B3) Fluorescence is depleted from an area that stretches from the muscle tip to the centrally located nuclei after bleaching, whereas fluorescence loss is limited in the muscle centre and in the other muscle end. (B4) Bar graph shows that fluorescence is mostly removed from the bleach spot (pink) and that fluorescence outside the bleached domain (yellow) is slightly diminished. (C1–C4) Myofibers expressing GFP-tagged mRNA (bleaching in the muscle centre). (C3) Fluorescence is depleted from the central area of the cell and remains present at both sides of the muscle. (C4) Bar graph shows the lack of fluorescence at the bleach spot (pink) and a slightly diminished fluorescence outside the bleached domain (yellow). (B3,C3) The areas surrounding the bleach spot from which the fluorescence was depleted will be referred to as bleached domains and are indicated with pink dashed lines. (D1–D4 and E1–E4) Myofibers expressing GFP. Fluorescence is lost throughout the cell after photobleaching at the muscle end (D3) or muscle centre (E3). Arrow points to an underlying myofiber in which the fluorescence was bleached away in the course of the FLIP experiment. (D4, E4) Bar graphs illustrate the distribution of fluorescence in the myofiber after bleaching.

To determine if the movement of GFP-tagged mRNAs is restricted within myofibers we performed a series of Fluorescence Loss In Photobleaching (FLIP) experiments. FLIP is a technique in which fluorescence is repeatedly bleached at one site within the cell and the loss of fluorescence is monitored throughout the cell. Using this approach all the fluorescently tagged molecules that are capable of moving into the area that is being photobleached will be bleached away over time. This technique allows the visualisation of isolated compartments and immobilised proteins in the cell [19]. Experiments were performed in muscle fiber 12. This muscle contains two clusters of nuclei that are situated around the central area of the cytoplasm. Due to the curvature of the embryo the entire muscle is not always present in the focus plane and one of the muscle ends is frequently not visible (Fig. 2C1). FLIP experiments span a total of 185 seconds and start with 2 pre-bleach images followed by 21 cycles composed of ten bleach events (8 s which includes bleaching and switching of the microscope from image to bleach mode and back again) and two images (772 ms). Bleaching was performed in an area of 1 µm in diameter located either at the muscle end, between the muscle tip and the centrally located nuclei, or in the muscle centre, in the cytoplasmic region in between the nuclei. To ensure that bleaching and quantification of fluorescence did not take place inside a nucleus we used a Hoechst nuclear staining to determine the location of the nuclei (Fig. 2A2–E2).

To establish the bleach parameters for these FLIP experiments we determined the effective bleach area using fixed embryos expressing GFP diffusely present throughout the muscle using FLIP settings as described above. With a circular bleach area of 1 µm in diameter, we observed an effective bleach area of approximately 4 µm in diameter (Supplemental Figure S1). The observation that the effective bleach area was larger than the user defined bleach spot can be the result of the geometry of the laser beam and has been previously discussed [20], [21]. However, the extent to which the effective bleach area exceeded the user defined bleach area was unexpected. To examine if diffusion of GFP due to incomplete fixation also contributed to the large effective bleach area we performed additional FLIP experiments on embryos that were fixed over a longer period of time. These experiments showed comparable effective bleach areas indicating that the large effective bleach area did not result from residual GFP movement (data not shown). We subsequently analyzed the effective bleach area after each bleach cycle and found that the diameter of the bleach area increased with the number of cycles (data not shown). This led us to speculate that light scatter, which results from the thickness of the embryo and from the heterogeneity of the tissues that make up the Drosophila embryo, contributes to the gradual increase in bleach area.

Although the relatively large effective bleach area might not be fully explained, we conclude that any variation in fluorescence intensity in live embryos outside the effective bleach domain after FLIP is an indication of movement of GFP-tagged molecules. The FLIP protocol used includes the acquisition of images and as a result an overall diminished fluorescence is observed in addition to the loss of fluorescence from the effective bleach area.

The mobility of GFP-tagged mRNA was first compared to GFP-tubulin which forms relatively immobile structures within myofibers. In GFP-tubulin-producing flies, GFP-tubulin is incorporated into microtubules and these microtubules do not move within the cell although dynamic assembly and disassembly of tubulin subunits at the microtubule ends probably does occur. After the FLIP routine, a bleached area with a diameter of 4 µm was found (Fig. 2A3). This area corresponds to the effective bleach area in our fixed embryo experiments and suggests that a large part of GFP-tubulin molecules were immobile during the time frame of the experiment. However, loss of fluorescence in the myofiber outside the effective bleach area (Fig. 2A4) suggested that part of the GFP-tubulin is not incorporated into microtubules and is free to move within the myofiber.

To determine the mobility of GFP-tagged mRNA throughout the myofibers we used the same FLIP protocol at the muscle end (Fig. 2B1) and found that fluorescence was lost from an area that exceeded the effective bleach domain (Fig. 2B3). The area devoid of fluorescence stretched from the muscle tip to the centrally located nuclei while fluorescence levels remained relatively high in the muscle centre and at the other side of the muscle. Upon repetitive photobleaching in the cytoplasm of the muscle centre (Fig. 2C1), fluorescence in the central area of the myofiber showed a substantial drop, compared with the slight drop in fluorescence in the cytoplasm at either side of the myofibers (Fig. 2C3). The muscles presented in figure 2 express a substantial amount of GFP- fusion protein resulting in accumulation of GFP in most nuclei. This allowed the best visualization of the domains revealed by the FLIP routine. However, the same domains are found when imaging myofibers expressing lower levels of GFP-fusion protein that contain many nuclei devoid of GFP (Supplemental Figure S2). Quantification of the fluorescence in the bleach spot (10–14% of fluorescence remaining) and in the cytoplasm outside the bleached area (71–75% of fluorescence remaining) illustrated the inhomogeneous distribution of fluorescence after FLIP (Fig. 2B4, C4).

Only a small loss of fluorescence was observed in most nuclei of the myofiber in which FLIP was performed. This was expected as the GFP-fusion protein residing in the nuclei represents the GFP-fusion protein that has not bound reporter mRNA and therefore remains present in the nucleus. Significant fluorescence loss in the nuclei was only observed when the nuclei were located within the effective bleach area as often occurred when photobleaching was performed in the muscle centre (Fig. 2C1, - C3).

Altogether these findings clearly showed that the movement of GFP-tagged mRNAs in myofibers is restricted and indicated that the cytoplasm of muscle 12 consists of three domains: two domains located at either ends of the myofiber and one central domain. These domains coincide with the areas within the cell from which the majority of the fluorescence is lost during FLIP experiments and constitute regions in the cell in which the GFP-tagged mRNA moves rapidly.

### Myofibers are not compartmentalised: GFP is free to move throughout the cell

As the domains we found were separated from one and other by the centrally located nuclei, we next asked whether these nuclei might act as physical barriers within muscle 12. To test this, we performed a comparable set of FLIP experiments in myofibers expressing GFP (Fig. 2D,E). GFP is not supposed to bind to any structures present in myofibers and should be able to move all through the muscle if the nuclei do not hinder movement from one side of the muscle to the other. We found a substantial drop in fluorescence throughout the entire muscle upon repetitive photobleaching either in the muscle end or muscle centre (Fig. 2D3, E3). Fluorescence in the bleach spot and in the domain adjacent to the bleached domain was quantified before and after photobleaching. The fluorescence remaining at the bleach spot after repetitive photobleaching at the muscle end amounted to 29% while 41% of the fluorescence remained present in the cytoplasm of the central domain (Fig. 2D4). A slightly non-uniform distribution of fluorescence was also found after repetitive photobleaching in the muscle centre, with 19% of the fluorescence remaining at the bleach spot and 32% at the muscle end domain (Fig. 2E4).

GFP is present in the cytoplasm and in the nuclei of the myofibers. In all FLIP experiments, GFP fluorescence was initially depleted from the cytoplasm (Movie S1) but during prolonged bleaching, exchange of GFP between the cytoplasm and the nucleus occurred and fluorescence was lost from the nuclei as well. As a result we also observed myofibers that had retained some GFP in the nuclei after the FLIP experiment (not shown for muscle 12). This indicated that GFP moves mostly around the nuclei although movement through the nuclei is likely to occur as well.

Close examination of the post-bleach images showed that the remaining fluorescence outside the bleach point often originated from underlying myofibers, which were visible because of the relatively large confocal pinhole size that had to be used as explained in Materials and Methods. In addition, myofibers located under the myofiber in which the FLIP experiments were performed, were also bleached if they were situated within the effective bleach volume (Fig. 2D3, arrow). This frequently occurred as the spatial organization of the muscles in each segment is conserved and underlying muscles are always found at their same stereotypic positions. Together, these results indicated that GFP protein moves freely throughout the cytoplasm of the myofiber and suggested that *Drosophila* body wall myofibers in themselves are not compartmentalized. These data further suggested that the slightly non-uniform distribution of the fluorescence found after FLIP does not result from restricted movement of GFP, but from the fluorescence present in underlying myofibers.

### Restricted movement of GFP-tagged mRNAs also found in muscle 8

**Figure 3:**
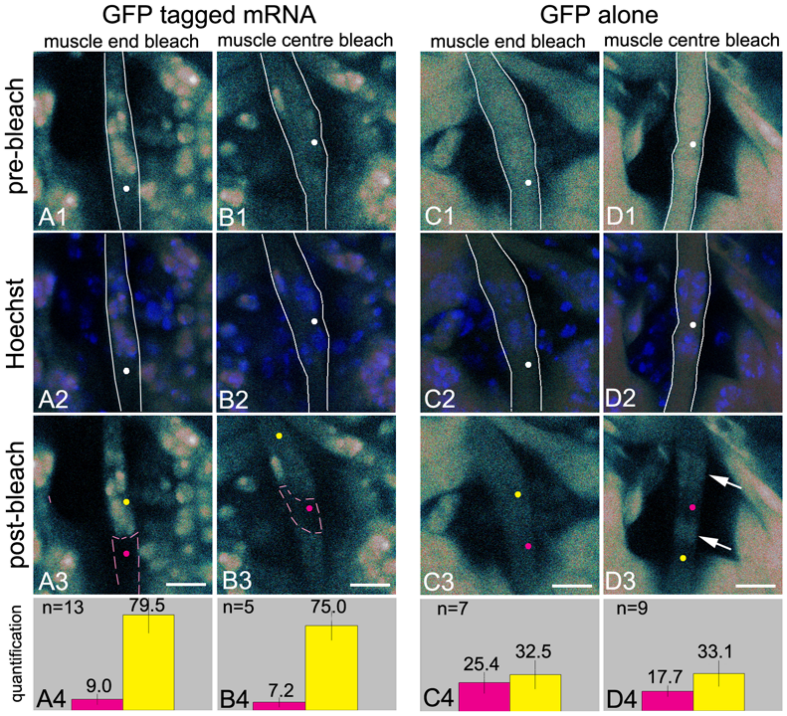
GFP-tagged mRNA movement in muscle 8 confirms the presence of myofiber domains. (A1–D1) Pre-FLIP confocal images. (A2–D2) Overlay of the pre-FLIP images and confocal images showing Hoechst stained nuclei, confirming that the bleach spot and quantification areas are chosen outside of the nuclei. (A3–D3) Post-FLIP confocal images. Bars, 8 µm. (A4–D4) Quantification. Information on white lines, white, yellow and pink dots, pink dashed lines and bar graphs are given in the legend of Fig. 2. (A1–A4) Myofibers expressing GFP-tagged mRNA (bleaching in the muscle end). (A3) Fluorescence is depleted from the lower muscle end and only slightly decreases in the muscle centre and in the upper muscle end. (A4) Bar graph confirms that fluorescence is removed from the bleach spot (pink) and remains mostly present in the domain adjacent to the bleached domain (yellow). (B1–B4) Myofibers expressing GFP-tagged mRNA (bleaching in the muscle centre). (B3) Fluorescence is depleted from the central domain and almost remains present in the two muscle end domains. (B4) Bar graph shows the lack of fluorescence at the bleach spot (pink) and a slightly diminished fluorescence in the domain adjacent to the bleached domain (yellow). (C1–C4 and D1–D4) Myofibers expressing GFP. (C3, D3) Fluorescence is lost throughout the cell after photobleaching at the muscle end or muscle centre. Arrow points to fluorescence remaining in the nuclei. (C4, D4) Bar graphs illustrate the relative homogeneous distribution of fluorescence in the myofiber cytoplasm after bleaching.

To investigate if myofiber domains can also be visualized in other muscles, we performed identical experiments in muscle 8. Muscle 8 is thinner than muscle 12, but like muscle 12, it contains a number of nuclei that are centrally located. Repetitive bleaching at the muscle end or in the centre of the cell outside the nuclei (Fig. 3A2, B2) showed substantial loss of fluorescence from the domains in which the bleaching was performed and only a small loss of fluorescence in the remainder of the cell (Fig. 3A, B). Quantification of fluorescence intensity in the bleach spot and in a neighbouring domain confirmed the inhomogeneous distribution of GFP-tagged mRNA after repetitive bleaching (Fig. 3A4, B4).

As found in muscle 12, GFP alone was free to move throughout muscle 8 (Fig. 3C, D). This resulted in the loss of fluorescence throughout the cell cytoplasm after repetitive bleaching of a single spot (Fig. 3C4, D4). Note that the partial retention of fluorescence in the nuclei after FLIP described for muscle 12 is now shown in muscle 8 (Fig. 3D3, arrows). These data clearly show that muscle 8 also features two muscle end domains and a central domain. As in muscle 12, the muscle end domains run from the muscle tips to the centrally located nuclei and the central domain contains the central area of cytoplasm that is surrounded by nuclei.

### Most mRNAs are free to move within the domains but a small fraction of the mRNAs located in the central domain is immobile

Given our observation that GFP-tagged mRNA was free to move within the domains outlined by the FLIP experiments, we next investigated the kinetic properties of the tagged mRNAs. To this end we performed Fluorescence Recovery After Photobleaching (FRAP) experiments in the muscle end and muscle centre domains. In FRAP experiments, fluorescence is photobleached once in a small area of the cell after which recovery of fluorescence in the same area is monitored over time. Using this approach the dynamic properties of fluorescent molecules can be determined [22].

**Figure 4:**
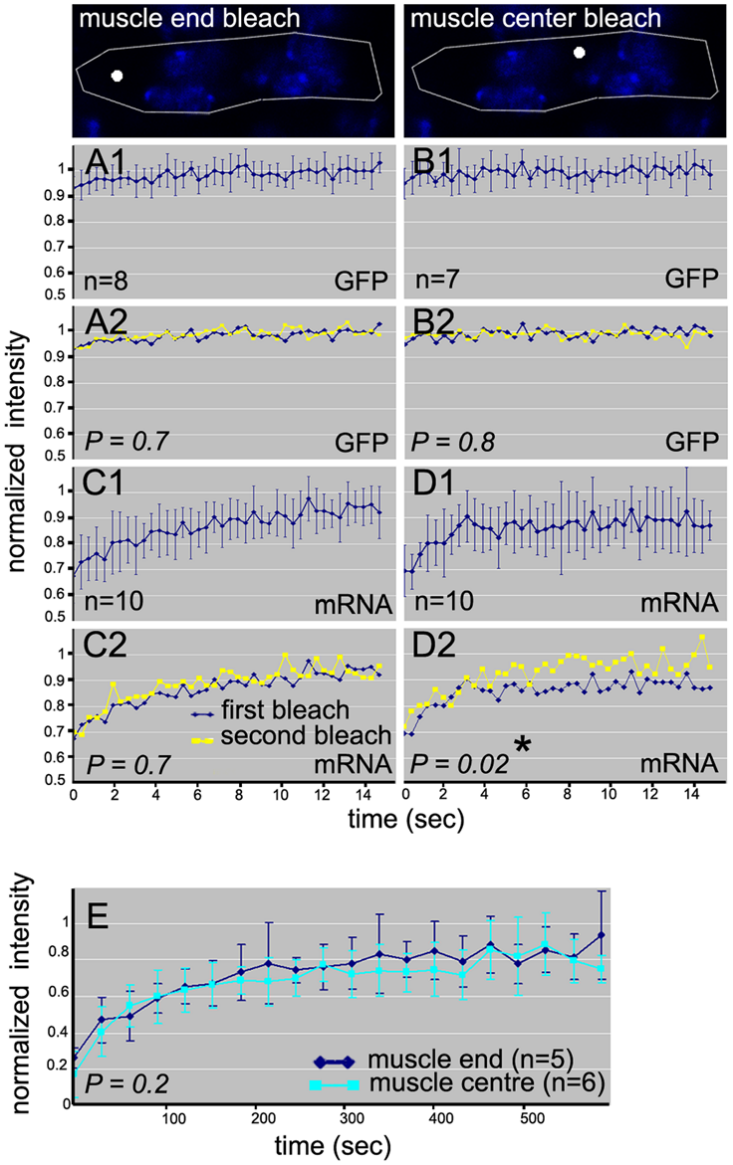
GFP-tagged mRNAs are mobile within the domains but exhibit limited exchange between domains. Confocal images of Hoechst stained nuclei. White lines show circumference of the muscle and white spots indicate bleach spot location. Error bars give standard deviation. Statistical analysis was done by paired-sample T-test with two-tailed distribution. * indicates significant difference between areas under the curve. *P*<0.05 is considered significant. (A1, A2, B1, B2) FRAP and double FRAP experiments in muscle 12 expressing GFP. (A1, B1) Fluorescence recovery was too fast to measure after bleaching at the muscle end or centre. (A2, B2) First and second recovery curves are comparable. (C1, C2, D1, D2) FRAP and double FRAP experiments in muscle 12 expressing GFP-tagged mRNA. (C1, C2) Almost full recovery after bleaching at the muscle end. Recovery curves are comparable after the first and second bleach. (D1) Fluorescence does not fully recover after bleaching at the muscle centre. (D2) Fluorescence recovers to 100% after the second bleach. Recovery curves after the first and second bleach show a significant difference indicating the existence of an immobile fraction. (E) Fluorescent recovery after FLIP in the muscle centre or muscle end in embryos expressing GFP-tagged mRNA in muscle 12 takes place.

After photobleaching an area in the muscle end we found a rapid and almost complete recovery of GFP-tagged mRNA fluorescence into the bleached area after 15 seconds (Fig. 4C1). Interestingly, when FRAP experiments were performed in the centre of the cell, in the area surrounded by nuclei, the recovery was not complete (Fig. 4D1) and fluorescence intensity seemed to have reached a plateau after 5 seconds already. This suggested that a subpopulation of the GFP-tagged mRNAs was relatively immobile in this area during the experiment.

We used a double-FRAP approach to test this [22]. In double FRAP experiments the first FRAP experiment is followed by a second experiment in which the same area is photobleached again. Immobile molecules that are photobleached during the first bleach will remain present in the photobleached area, while the mobile molecules are capable of exchange during the recovery period. This results in an incomplete recovery of fluorescence into the bleached area. During the second bleach only the mobile fraction will be bleached as the immobile fraction has remained non-fluorescent. Incomplete recovery after the first bleach followed by a complete recovery after the second bleach is therefore a clear indication of the presence of an immobile fraction.

Double FRAP experiments were performed at the muscle end and in the muscle centre. The incomplete recovery after the first bleach at the muscle centre (Fig. 4D1) was followed by a complete recovery after the second bleach. Analysis of the data clearly showed that first and second recovery curves were statistically different (p = 0.02) (Fig. 4D2). Double FRAP experiments in the muscle end showed an almost full recovery after both the first (Fig. 4C1) and second bleach with comparable first and second recovery curves (p = 0,7) (Fig. 4C2). These data showed that GFP-tagged mRNAs move within the myofibers and that a small immobile fraction is present in the central area of the cell. The presence of this small immobile fraction distinguishes the central cytoplasmic domain from the two muscle end domains.

We also determined the mobility of GFP alone. As expected for molecules that do not bind to structures within the cell, FRAP experiments showed that the bleached area was rapidly repopulated immediately after photobleaching (Fig. 4A1, B1). Indeed, the time the microscope hard- and software requires to switch between bleaching and the acquisition of the first post-bleach image is too long to visualize recovery. In addition, first and second bleach recovery curves for GFP were comparable (Fig. 4A2, B2). Hence, GFP is highly mobile and moves substantially faster than GFP-tagged mRNA.

### Limited exchange of GFP-tagged mRNAs between the domains

The diminished amount of fluorescence outside the bleached domain after FLIP suggested that mRNA movement between the domains might be possible in the time frame of our FLIP experiments. To further analyse the movement of GFP-tagged mRNAs in and out of these domains, the recovery of fluorescence after FLIP was monitored. These experiments showed that repopulation of the bleached domains required approximately 6 min, whether bleaching was performed in the muscle end or in the muscle centre. As expected the fluorescence intensity after FLIP never fully recovered to pre-FLIP levels (Fig. 4E) as a substantial part of the total initial fluorescence was bleached away during the FLIP procedure. These results showed that exchange of mRNAs between the domains occurs but is relatively slow. These data further suggested that the movement of GFP-tagged mRNA between the domains lacks directionality since the recovery of fluorescence into the muscle centre did not significantly differ from the recovery into the muscle end.

### Endogenous RNAs show restricted movement within myofibers

To determine if endogenous RNAs also respect the boundaries of the domains revealed by the movement of GFP-tagged reporter mRNA, we performed FLIP experiments in myofibers in which endogenous RNAs were labelled using E36. E36 is a small membrane permeable RNA-selective probe that has been previously used in live cell imaging. The quantum fluorescence yield of E36 increases 54 times after binding RNA and it is thought to recognize all RNA species [23]. To determine the binding kinetics of E36 to RNAs we bleached an entire myofiber and monitored the recovery over time. We found that fluorescence recovered over time (550 seconds) suggesting that bound and bleached E36 is released from the RNA and is subsequently replaced by other E36 molecules that are present in the embryo, thus allowing fluorescence recovery. As expected, no recovery of fluorescence was observed after photobleaching in myofibers expressing GFP and GFP-tagged mRNAs. We conclude that E36 is not tightly bound to RNA resulting in some exchange of bound probe during the FLIP experiments (Supplemental Figure S3).

**Figure 5:**
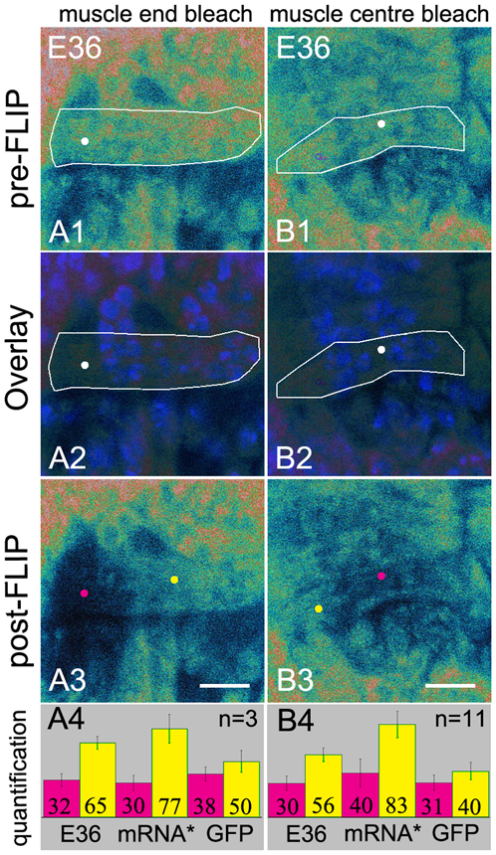
Endogenous RNAs are also limited with respect to their movement in myofibers. FLIP experiments in the muscle 12 of wild type *Drosophila* after injection of Hoechst and E36. (A1, B1) Pre-FLIP confocal images. (A2, B2) Overlays of pre-FLIP images and confocal images showing Hoechst stained nuclei confirming that the bleachspots are located outside the nuclei (white dots). (A3, B3) Post-FLIP confocal images. Bars, 8 µm. (A4, B4) Quantification. Information on white lines, white, yellow and pink dots and the bar graphs are given in the legend of Fig. 2. (A3) Bleaching at the muscle end. Fluorescence is lost from the muscle end but remains present in the rest of the cell. (B3) Bleaching at the muscle centre. Fluorescence is lost from the muscle centre and remains present at the both muscle ends. These data are compared with the FLIP data from GFP and the reporter mRNA expressing myofibers (Fig. 2) (A4, B4) Bar graphs show that the fluorescence in the bleached spots (pink) is reduced and that the fluorescence outside the bleached domains (yellow) is also diminished but to a lesser extent than when FLIP experiments were performed in GFP expressing myofibers. mRNA* = GFP-tagged mRNA. For quantification of the fluorescence see Materials and Methods.

Repetitive photobleaching at the end or in the centre of muscle 12 showed a substantial loss of fluorescence in the domain in which bleaching was performed and in the nuclei that bordered the bleached domains. The fluorescence in the remainder of the cell was also affected but the drop in fluorescence was much less pronounced compared to the loss of fluorescence in the bleached domain (Fig. 5A2, A3, B2, B3). FLIP experiments in myofibers expressing GFP-tagged mRNAs only showed loss of fluorescence from the nuclei when the nuclei were located within the effective bleach area. Loss of fluorescence from the nuclei after repetitive photobleaching of E36 labelled RNAs therefore suggested the presence of an E36 bound RNA species moving in and out of the nuclei in the time frame of these experiments. The overall contribution of the movement of RNA species other than mRNAs in these experiments remains unclear. Also some recovery of fluorescence is due to the release of bleached E36 from endogenous RNA and subsequent renewed binding of previously unbound E36 during the experiment as described above.

We subsequently compared the loss of fluorescence after FLIP for E36 labelled endogenous RNAs with that observed in our previous–GFP and GFP-tagged mRNA- FLIP experiments (Fig. 5A4, B4). In E36 labelled myofibers, we found that 65% of the original fluorescence in the muscle centre remained after bleaching in the muscle end and that 56% remained at the end of the muscle after bleaching at the muscle centre. These values were only slightly lower than the values found after repetitive photobleaching of GFP-tagged mRNA at the muscle end or muscle centre (77% and 83% remaining respectively) (Fig. 5A4, B4). These data suggested that, although E36 labelled RNAs do not solely reflect the behaviour of mRNA, endogenous RNAs, similar to GFP-tagged mRNAs, are restricted with respect to their movement within the cell. However, the domains in which they are free to move are less well defined.

### Ultrastructural analysis of myofibers does not point to cellular structures that could make up the barriers between the domains

**Figure 6:**
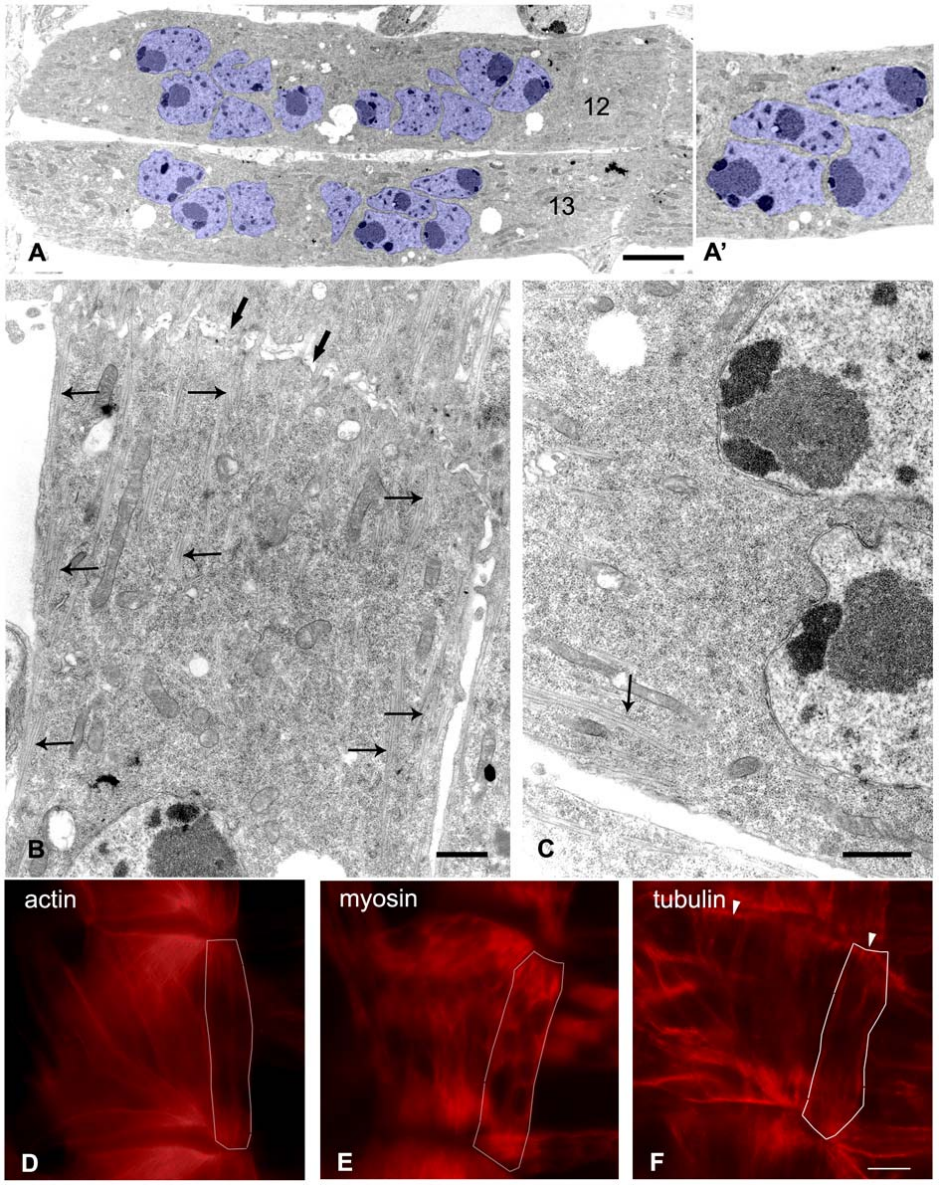
Exchange of mRNAs between the domains along the periphery of the myofiber. (A–C) Representative electron micrographs of VLM muscles 12 and 13. (A) Overview of muscle showing the location of the nuclei. (A') Note the exceptional close fit of the nuclei in blue. (B) Muscle end bordering on segment border. Thick arrows indicate segment border. Slim arrows indicate aligned actin and myosin filaments emanating from muscle tip and present under the plasma membrane. Note that the actin and myosin filaments become less prominent in the centre of the cell close to the nuclei. (C) Actin and myosin are present along the border of the muscle (arrow) but absent from the centre of the cell. Bar A, 5 µm. Bar B, 1 µm. Bar C, 1 µm. (D–F) Fluorescent staining of actin, myosin and tubulin. White lines indicate the outline of muscle 12. All cytoskeletal elements were present along the plasma membrane and are less prominent in the centre of the cell. (D, E) Myosin and to a lesser extent actin are abundant at both ends of the muscle. (F) Tubulin shows most pronounced staining at the segment borders (arrowheads). Bar F, 5 µm.

Given our observation that the domains visualized by the movement of GFP-tagged mRNA are sharply delineated and always separated from the adjacent domain(s) by nuclei, we asked what the role of the nuclei in domain formation might be. The FLIP experiments in GFP-expressing myofibers showed that the nuclei do not prevent movement of GFP through the cell. To further examine the role the nuclei might play we investigated the ventral longitudinal muscles (VLM) including muscle 12 by electron microscopy (EM). We found that the centrally located nuclei can be in very close proximity to each other (Fig. 6A, A', nuclei labeled in blue). Indeed nuclei that are located in close proximity often show indentations and complementary protuberances that allowed the nuclei to assume an exceptionally close fit (Fig. 6A'). Transport through the central part of the muscle, through the area in which the nuclei are in such close apposition could therefore be limited. The central nuclei are, however, located at a substantial distance from the plasma membrane (Fig. 6A) resulting in a sizeable area at the periphery of the myofiber through which diffusion of macromolecules should be possible. It thus seems likely that the exchange of macromolecules through the centre of the myofibers occurs predominantly in the area located between the nuclei and the plasma membrane.

Further ultrastructural investigation of the myofibers did not reveal other structural components that might contribute to the boundaries of the domains. Indeed rough endoplasmic reticulum membranes which are typically found surrounding nuclei were notably absent from the perinuclear cytoplasm in body wall myofibers (Fig. 6B, C). Taken together these data suggested that the domains may not be separated by true physical barriers but might result from transient or prolonged associations of the GFP-tagged mRNAs to structures in the cytoplasm located close to the nuclei.

### Microtubules at the periphery of the myofibers might contribute to the maintenance of the domains

Double-FRAP experiments showed that a small but significant immobile fraction is present in the centre of the cell and recovery after FLIP further indicated that exchange of the mRNAs between the domains is relatively slow. Together these data suggested that the mRNAs might undergo more prolonged association with cellular components in the centre of the myofiber.

As it has been reported that mRNAs and ribosomes often associate with cytoskeletal elements [4], [24] we examined these structures in the myofibers with particular focus upon the cell centre. The electron micrographs showed clearly visible alignments of actin and myosin fibers that emanate from the muscle tips (Fig. 6B, arrows). They only sporadically entered the central area of the cell except where they were present immediately under the plasma membrane (Fig. 6C, arrow). Immunofluorescent stainings of actin and myosin (Fig. 6D, E) were in agreement with these findings. These stainings showed that actin and myosin are present in the muscle end and underneath the plasma membrane in the middle of the cell. Myosin is particularly abundant at the muscle end (Fig. 6E).

Microtubules were difficult to discern in the electron micrographs, however, immunostaining of tubulin revealed that microtubules are prominently present along the entire outline of the VLM myofibers (Fig. 6F). Strong anti-tubulin staining was found at the segment border where the VLM muscle tips make contact with the tendon cells (Fig. 6F arrowhead) while substantially less staining was found in the muscle end in comparison to both the myosin and actin stainings. We found that GFP-tagged mRNA was free to move throughout the muscle end, suggesting that these mRNAs do not extensively interact with myosin and actin filaments that are abundant in this area of the cell. Therefore, microtubules present at the periphery of the myofibers might be involved in maintaining the slow exchange of mRNAs between the domains.

To determine the contribution of microtubules in domain maintenance, we attempted to analyse mRNA movement after injecting a microtubule depolymerization agent into *Drosophila* embryos. This procedure was successful when studying the involvement of microtubules in the compartmentalization of the endoplasmic reticulum in early embryos [25]. In our hands, however, microinjection of nocodazole at embryonic stage 13 did not result in the disruption of the microtubule network (data not shown). The lack of effect of the nocodazole treatment might be due to the presence of stable, destabilizing drug resistant, microtubules that have been described in differentiating mammalian myofibers [26], [27] and in *Drosophila* oocytes [28].

## Discussion

**Figure 7:**
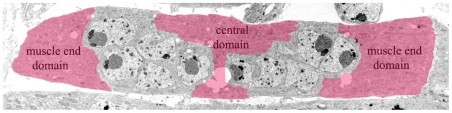
Schematic representation of the domains visualized in this study. Two muscle end domains and the central domain given in red are superimposed on electron micrograph of muscle 12. The domains are defined as areas from which the fluorescence is lost in the course of the FLIP experiment due to the mobility of the mRNAs resulting from the low affinity of the GFP-tagged mRNAs for the binding sites in the domain or the absence of binding sites. The borders of the domains are positioned at some distance from the nuclei thus taking into account the putative limited movement around the nuclei due to the prolonged association of the mRNAs to up to now undefined structures present close to the nuclei.

In this study we used transgenic lines expressing GFP-tagged mRNAs to visualize the movement of reporter mRNAs in the large multinuclear myofibers present in the body wall of *Drosophila* embryos. We found that these myofibers consist of three domains and that the reporter mRNAs move freely within the domains but that exchange between the domains is relatively slow. These domains are located at the centre and at either end of the myofiber. In Fig. 7 we have superimposed the domains described in this paper on the micrograph of muscle 12. The domains are specific for the GFP-tagged mRNA as GFP alone was free to move throughout the myofiber.

We characterized the movement of the reporter mRNAs within the domains using FRAP methods. We found that the reporter mRNAs are highly mobile within the domains outlined in this study, but that GFP alone moves substantially faster as indicated by the nearly instantaneous recovery after photobleaching in the FRAP experiments. The mobility of molecules within a cell depends on both the diffusion and the binding dynamics of these molecules. Therefore the rapid recovery of GFP was in agreement with the assumption that GFP does not interact with cellular structures in the myofibers and with the observation that the half-time for FRAP recovery of the 27 kDa GFP is only 0.011 sec [29].

The GFP-tagged mRNA contains eight MS2 binding sites that can each bind a maximum of two (45 kDa) GFP-fusion proteins. The protein mass of this complex therefore can not exceed 720 kDa. As FRAP recovery rates due to diffusion are only weakly dependent on protein mass [29] these data suggested that GFP-tagged mRNAs undergo interactions with cellular components in the cytoplasm. In this context two types of interactions have been described: 1] mRNAs are mostly part of messenger ribonucleoprotein particles (mRNPs) [30], [31] and depending on the fate of the constituent mRNAs, these mRNPs can in turn become part of even larger structures such as P-bodies, stress granules, or polysomes. 2] In addition mRNAs, mRNPs and ribosomes are reported to interact with cytoskeleton elements [4], [5], [31].

Our EM findings suggested that the slow exchange between the domains primarily takes place between the plasma membrane and the centrally located nuclei but did not reveal structures that might contribute to the domain border. We found that microtubules are abundantly present immediately underneath the myofiber plasma membrane but it proved impossible to disrupt these stable microtubules and we could not determine the involvement of these structures in domain formation. Interestingly in oocytes restricted movement of *grk* mRNA appears to coincide with the appearance of drug resistant microtubules, suggesting that stable microtubules are involved in mRNA anchorage [28].

Our finding that a small immobile fraction of mRNAs was present in the central domain in the area surrounded by nuclei also pointed towards the involvement of structures located in close proximity to the nuclei. Recently outer nuclear membrane proteins containing a conserved KASH domain have been described that are involved in nuclear positioning within large multinucleated cells. In *Drosophila* the KASH domain containing protein MSP-300 plays an important role in positioning nuclei in egg chambers [32] and mice lacking the KASH domain protein Syne-1, display mislocalized nuclei in skeletal muscle [33]. KASH domain proteins are therefore interesting potential candidates well suited to play a role in domain maintenance and will be a subject of further investigation.

The implications of this general restrictive mechanism for specific mRNAs that do contain signalling sequences are not addressed in this paper. Applying the recently developed method that uses the MS2 system to label endogenous mRNAs could prove an elegant approach to answer this question in the future [28].

The finding that myofibers are compartmentalized and consist of domains was not altogether unexpected. In adult myofibers nuclei are evenly spaced along the entire length of the fiber and each nucleus is surrounded by a volume of cytoplasm that is nurtured by the gene products derived from this nucleus [34]. At a protein level, the restricted movement of mRNAs is reflected in a non homogeneous distribution of some proteins in hybrid myotubes [35]–[37].

Our work was performed in young fibers at a stage in which the myofibers do not yet contract and the nuclei are still located at the centre of the cell. The domains we describe in these young fibers differ from the nuclear domains described in fully differentiated myofibers in that they are not associated with a single nucleus but are, in fact, bounded by nuclei. Further study will be necessary to clarify the possible relationship between the domains in the adult myofibers and the domains that we for the first time describe in developing myofibers.

Understanding the movement of mRNAs within myofibers is important as it has consequences for regenerative therapies that are currently being developed to treat myopathies [38]. These therapies aim to change an entirely diseased myofiber into a hybrid myofiber that consists of both healthy and diseased nuclei. Cell-based therapies, as described above, might have a limited effect if the mRNAs transcribed by the healthy nuclei and the proteins they encode are restricted to their nuclear domain and cannot redistribute throughout the myofiber. In the future, we hope to employ this *Drosophila* model system to shed light on the mechanisms that restrict mRNA movement in myofibers and possibly find ways to manipulate these mechanisms.

## Materials and Methods

### Transformation plasmids

Constructs were generated using the pUAST vector [15] in which an Age1 site was introduced into the multiple cloning site (pUAST-AgeI).

The reporter mRNA construct UAS-lacZ-MS2bs was made by excising part of the lacZ-8 MS2bs from the RSV-lacZ-MS2bs plasmid [39] with AgeI and BglII and inserting it into pUAST-AgeI in between the AgeI and BglII site. This construct (UAS-lacZ-MS2bs minus 5′) lacked the first 120 bp. This 5′ sequence with flanking Age1 sites and a *Drosophila* translation initiation site consensus sequence [40] was generated by PCR using RSV-lacZ-MS2bs as template and the following primers: LacZAge 5′-ATATAACCGGTGCTAGCCAAAACATGAGCGAAAAATACATCG-3′. LacZ fev 5′-GGGTTGAATTAGCGGAACG-3′.

The PCR fragment was ligated in a pGEM-TEasy vector (Promega Benelux B.V., Leiden, The Netherlands). This plasmid, was cleaved with AgeI and the resulting fragment was ligated into AgeI cleaved UAS-lacZ-MS2bs minus 5′.

The GFP-fusion protein construct UAS-GFP-MS2-nls was generated by excising GFP-MS2-nls from the CMV-GFP-MS2-nls vector [39] with AgeI and KpnI and ligating in between the AgeI and KpnI sites of pUAST-AgeI. RSV-lacZ-MS2bs and CMV-GFP-MS2-nls vectors were kind gifts from Dr. Kosik.

### Drosophila stocks

Wild type line was w^1118^. The UAS-EGFP (B-5431) and UAS-GFPS65C-alphaTub84B (B-7374) [41]. Small stocks were obtained from the Bloomington *Drosophila* Stock Center. The 24B-Gal4 driver line [15] was used for expression in the somatic muscles of stage 16 embryos.

### Transgenic Drosophila stocks expressing GFP-tagged mRNA

Transformation constructs described above were used to generate transgenic flies. Transformants with DNA inserts on the second, third and X-chromosome were obtained. Transformants varied in expression level. From the UAS-lacZ-MS2bs independent homozygous fly founder lines obtained, the highest expressor was selected for the experiments. From the UAS-GFP-MS2-nls independent homozygous fly founder lines obtained, the lowest expressor on the X-chromosome was selected for the experiments. To analyze GFP-tagged reporter mRNA in somatic muscles of stage 16 embryos the following crosses were set up: 24B-Gal4 males and UAS-GFP-MS2-nls females were crossed. Female offspring were crossed with UAS-lacZ-MS2bs males.

### Microinjection of live embryos

Embryos were collected for 5 hours at 21°C on apple juice agar plates, aged for 17 hours at 18°C, dechorionated in 50% bleach, lined up on slides using heptane glue, dehydrated for 14 minutes over silica gel and covered with Halocarbon oil (Halocarbon, New Jersey, U.S.). Microinjection was performed at stage 13 following standard techniques. Hoechst 33342 (Sigma-Aldrich, Zwijndrecht, The Netherlands) was microinjected in the posterior end at 10 mg/ml in injection buffer. E36 (a kind gift from Dr. Y.T. Chang) was injected into wild type embryos at 2.5 mM in injection buffer and Hoechst 10 mg/ml. Embryos were further aged at 18°C for at least two hours before imaging.

### Fixation of embryos

Fixation of the embryos [42] used to determine the bleach spot size after FLIP involves a 20 min incubation with 1.8% acid free formaldehyde. To ensure that the relatively large bleach spot did not result from residual diffusion due to incomplete fixation we also fixed the embryos for a longer period of time, 45 min using 1.8% formaldehyde.

### Imaging living embryos using laser confocal microscopy

To diminish the pressure on the embryos during the analysis three 18×18 mm coverslips were attached to the slide with heptane glue, surrounding the lined up embryos. A 24×50 mm coverslip was subsequently positioned on top of the coverslips. All experiments were carried out on a Leica TCS SP5 DMI6000 confocal microscope (Leica Microsystems, Wetzlar, Germany) (HCX PL APO 63×/1.4 NA oil-immersion objective, 12 bit resolution, 1024×1024 pixels, 1400 Hz speed, pinhole 3.1 Airy discs, zoomfactor 6) at room temperature for a maximum of 1 hour.

The embryos investigated expressed low levels of GFP-fusion protein to prevent excessive accumulation of GFP in the nucleus over time. In addition embryos are surrounded by a vitelline membrane and the muscles under investigation are located 17 µm underneath the surface of the embryo and light is therefore partly scattered before it reaches the myofibers. To compensate for the weak signal and energy loss due to light scattering we chose to work with a relatively large pinhole. This is essential for fast image sampling and the minimization of both focal drift and photobleaching due to scanning [43]–[45].

GFP images are shown in pseudocolors (Leica Application Suite-Advanced Fluorescence software (LAS-AF)). Photobleaching was performed using the 488 nm line from an Argon laser (measured through the 10× objective) operating at ∼3,8 mW, collecting emission between 500 and 600 nm. Nuclei stained with Hoechst were imaged with the 405 nm diode, collecting emission between 410 nm and 580 nm.

To verify that experiments were performed under physiological conditions the survival of the embryos after imaging was determined. Embryos were allowed to further develop after undergoing a standard imaging procedure. Most embryos (97%) developed into larvae and a majority formed pupae and developed further into flies.

### FLIP protocol

Nuclei were stained with Hoechst prior to imaging making it possible to position the bleach spot outside nuclei. FLIP experiments span a total of 185 seconds and start with two pre-bleach images (minimal frame scanning time 386 ms per image) (acquired with ∼0,57 mW) followed by a loop of 21 cycles composed of ten bleach events (8 s which includes bleaching and switching of the microscope from image to bleach mode and back again) of a 1 circular (diameter 1 µm) Region Of Interest (ROI) (∼3,8 mW, zoomfactor 64) and two images (772 ms). Movie was generated using the first image of each cycle.

Fluorescence recovery after FLIP was monitored by taking 20 images after the FLIP experiment (∼0,57 mW) at 30 s time interval. Loss of the fluorescence due to the recurrent scanning that is part of the FLIP protocol was never more than 5%.

Quantification of the fluorescence before and after repetitive photobleaching was performed in 2 circular areas with a diameter 1 µm in the myofiber: 1] an area that coincides with the bleach spot and 2] an area in the domain adjacent to the domain in which repetitive photobleaching was performed. Acquired data were analysed using LAS AF software. FLIP data were corrected for background signal (region chosen in an area devoid of any myofibers) and the percentage of fluorescence remaining within the bleach spot and the percentage remaining in the adjacent domain were calculated. Averages were plotted with a 1× standard deviation using Microsoft Office Excel 2003. Bar graphs give the averages of at least 6 FLIP experiments. Fluorescence intensities from E36 FLIP data were not corrected for background signal, because in some embryos no lower value than the post-bleach area could be found.

### FRAP protocol

Nuclei were stained with Hoechst prior to imaging making it possible to position the bleach spot outside nuclei. Each FRAP experiment starts with taking two pre-bleach images (772 ms) (∼0,38 mW) followed by bleaching of a 1 µm circular ROI (∼3,8 mW, zoomfactor 64) with a single scan and monitoring the recovery by taking 40 images (386 ms) (∼0,38 mW). For the double-FRAP experiment this session was repeated immediately (after approximately 5.7 seconds) with the same bleach ROI [22]. Special care was taken when doing these experiments not to include nuclei in the bleach ROI. Loss of fluorescence due to scanning during the FRAP protocol was never more than 12%.

Acquired data were analysed using LAS-AF software. To create FRAP curves, the fluorescence intensities were background-subtracted (region chosen in an area devoid of any myofibers), scan-corrected through dividing by the whole muscle intensity, and normalized to pre-bleach values [22]. Averages were plotted in Microsoft Office Excel 2003. To determine a significant difference between the recovery curves, the area under a curve (AUC) was analyzed by first restructuring data in SPSS 14.0 followed by computing the AUC in NCSS 2007. P-values were calculated with a paired-sample T-test with two-tailed distribution.

P-values of the recovery curves after FLIP were calculated with a two sample equal variance T-test. We examined the influence of bleach depth in analysis of covariance with a linear mixed model and a general linear uni variate model respectively (SPSS 14.0). Series were considered different when the resulting p-value was less than 0.05.

### Electronmicroscopy

Embryos were embedded and prepared for transmission electron microscopy as described previously [46]. More than 10 VLM muscles were analysed.

### Immunohistochemistry

Embryos were collected on apple juice plates at 21°C. Antibody labeling [42] and staging of embryos [47] was performed as described. Primary antibodies: rabbit-anti-ßGal (Cappel, Aurora, USA), mouse-anti-GFP 3E6 (Invitrogen, Breda, The Netherlands), mouse-anti-muscle myosin [48] (a kind gift from Dr. Corey Goodman) and mouse-anti-ß-tubulin (E7, Developmental Studies Hybridoma Bank (DSHB), Iowa). Secondary antibodies: HRP conjugated goat-anti-rabbit (Jackson Immunoresearch laboratories, Suffolk, UK), and AlexaFluor^568^ and AlexaFluor^488^-conjugated secondary antibodies (Invitrogen). AlexaFluor^568^-conjugated Phalloidin (1∶50) (Invitrogen) was used for actin staining. Embryos were mounted in Citifluor (Agar scientific Ltd., Essex, UK). Images were taken on a Leica DMRA fluorescence microscope, with a Photometrix quantix camera, a 100W mercury lamp, and a 100× NA1.3 plan Apo objective, using Colour Proc software [49]. Actin staining required an adapted protocol [48], [50]. Embryos were collected and staged as described in live embryo experiments but fixed for 5 minutes immediately after dechorionization.

## Supporting Information

Figure S1 The effective bleach area generated by the FLIP procedure is about 4 µm in diameter. (A) Pre-FLIP image taken of a fixed GFP expressing embryo. The user-defined bleached area (1 µm in diameter) is indicated by a white dot. (B) Post-FLIP image of the same embryo. The muscle was submitted to 20 cycles consisting of two images followed by ten bleach events. The effective bleach area indicated by a dashed line is approximately 4 µm wide. Scale bar: 8 µm. Muscles are outline in white.
(0.86 MB TIF)

Figure S2 Myofiber domains also revealed in muscles expressing lower levels of GFP-MS2-nls in their nuclei. (A1–A3) Muscle end bleach. (A1) Pre-FLIP image. Note that some nuclei are empty (arrow). (A2) Overlay of the pre-FLIP image with the Hoechst image indicating that the bleachspot was chosen outside of the nuclei at the right muscle end (white dot). (A3) Post-FLIP image. Fluorescence has dropped in the bleached domain whereas only a slight decrease is observed in the two other domains (arrowheads). (B1–B3) Muscle centre bleach. (B1) Pre-FLIP image. Empty nucleus marked by arrow. (B2) Overlay of the pre-FLIP image with the Hoechst image. The bleachspot is positioned in a region devoid of nuclei in the muscle centre (white dot). (B3) Post-FLIP image. Fluorescence has dropped in the central bleached domain whereas only a slight decrease is observed at the two muscle ends (arrowheads). Scale bar: 8 µm. Muscles are outline in white.
(2.80 MB TIF)

Figure S3 E36 dissociates from the RNA in the course of the FLIP experiments. To determine if E36 dissociates from the RNA during the FLIP experiments we bleached an entire myofiber and monitored the recovery of the fluorescence over time. In GFP and GFP-tagged mRNA expressing myofibers fluorescence did not recover as expected. In E36 injected embryos recovery over time was observed. Quantitative analysis showed 90% recovery of fluorescence after 550 seconds, with half the fluorescence recovered after 225 seconds. We conclude that excess unbound E36 is present in the myofibers and that E36 exchange takes place during the FLIP experiments (185s).
(4.47 MB TIF)

Movie S1 Loss of GFP fluorescence from cytoplasm and nuclei during FLIP experiment.
(0.31 MB MOV)
