# Supplementary figures and images for: In Vivo Monitoring of mRNA Movement in Drosophila Body Wall Muscle Cells Reveals the Presence of Myofiber Domains
Source: PLoS One. 2009 Aug 17;4(8):e6663. doi: 10.1371/journal.pone.0006663 (PMC2722729; doi:10.1371/journal.pone.0006663)

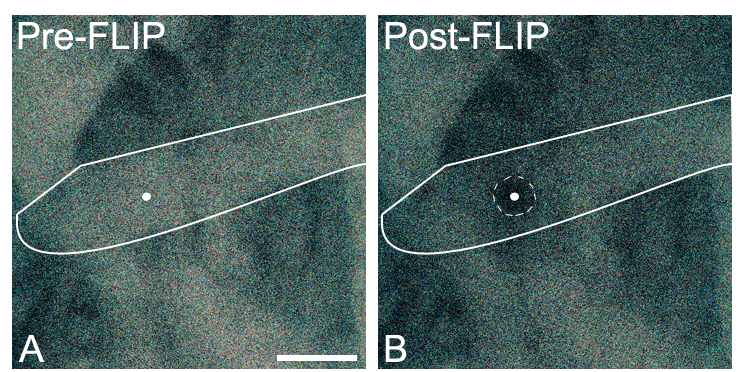

Supplement: Figure S1 — The effective bleach area generated by the FLIP procedure is about 4 µm in diameter. (A) Pre-FLIP image taken of a fixed GFP expressing embryo. The user-defined bleached area (1 µm in diameter) is indicated by a white dot. (B) Post-FLIP image of the same embryo. The muscle was submitted to 20 cycles consisting of two images followed by ten bleach events. The effective bleach area indicated by a dashed line is approximately 4 µm wide. Scale bar: 8 µm. Muscles are outline in white. (0.86 MB TIF) [file pone.0006663.s001.tif]

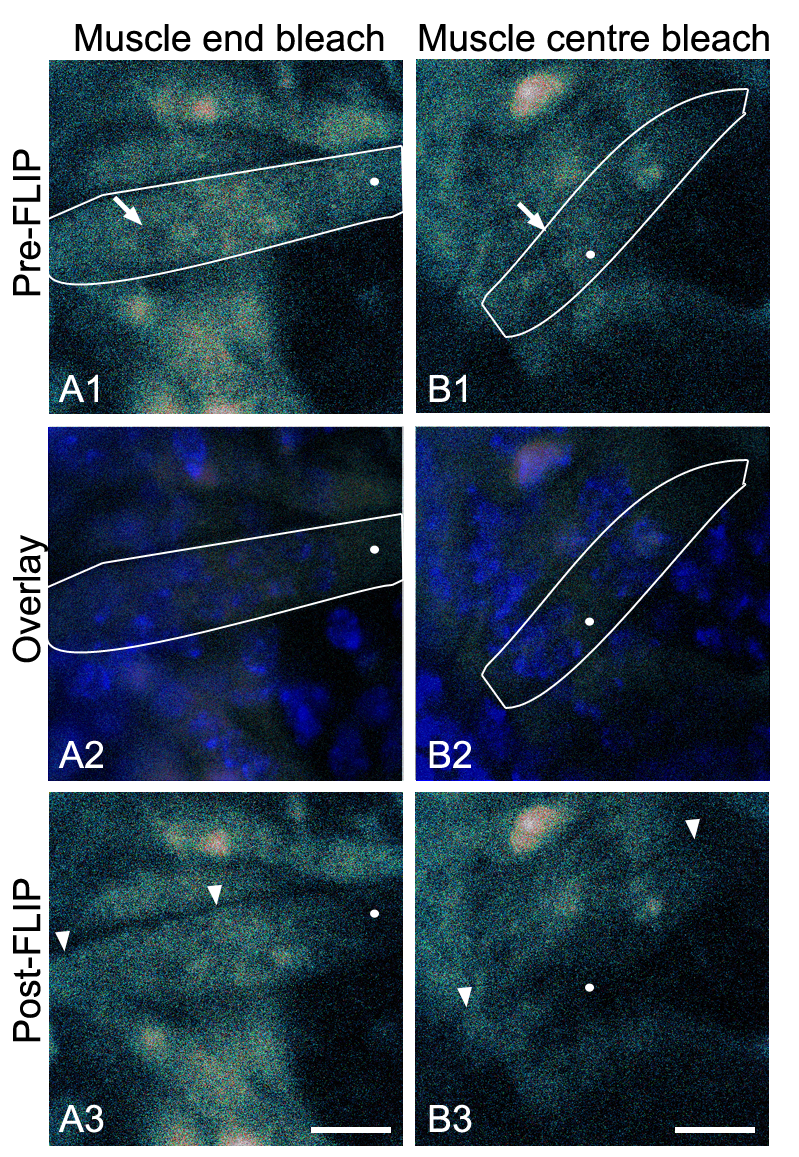

Supplement: Figure S2 — Myofiber domains also revealed in muscles expressing lower levels of GFP-MS2-nls in their nuclei. (A1–A3) Muscle end bleach. (A1) Pre-FLIP image. Note that some nuclei are empty (arrow). (A2) Overlay of the pre-FLIP image with the Hoechst image indicating that the bleachspot was chosen outside of the nuclei at the right muscle end (white dot). (A3) Post-FLIP image. Fluorescence has dropped in the bleached domain whereas only a slight decrease is observed in the two other domains (arrowheads). (B1–B3) Muscle centre bleach. (B1) Pre-FLIP image. Empty nucleus marked by arrow. (B2) Overlay of the pre-FLIP image with the Hoechst image. The bleachspot is positioned in a region devoid of nuclei in the muscle centre (white dot). (B3) Post-FLIP image. Fluorescence has dropped in the central bleached domain whereas only a slight decrease is observed at the two muscle ends (arrowheads). Scale bar: 8 µm. Muscles are outline in white. (2.80 MB TIF) [file pone.0006663.s002.tif]

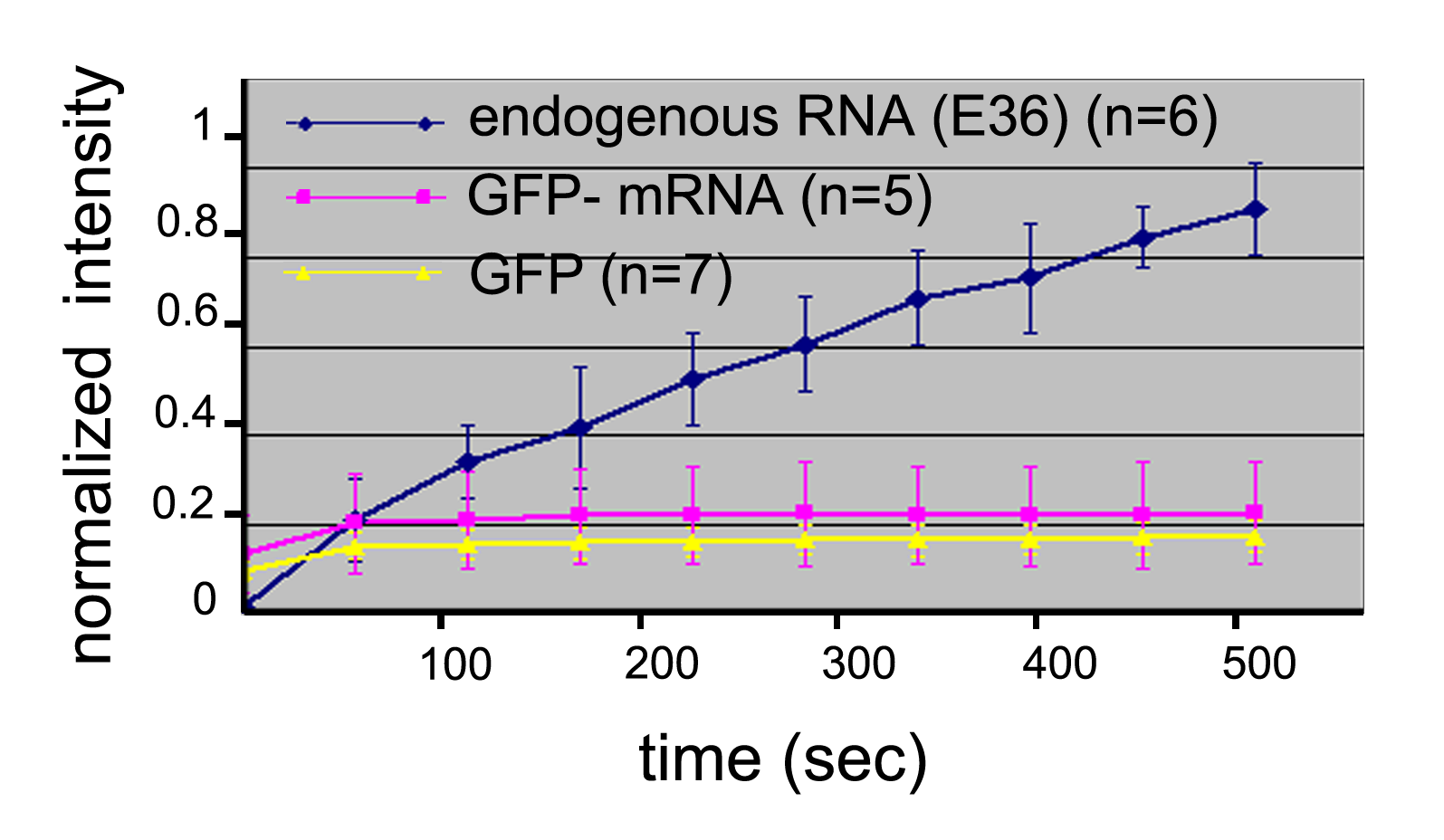

Supplement: Figure S3 — E36 dissociates from the RNA in the course of the FLIP experiments. To determine if E36 dissociates from the RNA during the FLIP experiments we bleached an entire myofiber and monitored the recovery of the fluorescence over time. In GFP and GFP-tagged mRNA expressing myofibers fluorescence did not recover as expected. In E36 injected embryos recovery over time was observed. Quantitative analysis showed 90% recovery of fluorescence after 550 seconds, with half the fluorescence recovered after 225 seconds. We conclude that excess unbound E36 is present in the myofibers and that E36 exchange takes place during the FLIP experiments (185s). (4.47 MB TIF) [file pone.0006663.s003.tif]
